# RAICEX: A Successful Story of the Spanish Scientific Diaspora

**DOI:** 10.3389/frma.2022.905765

**Published:** 2022-07-13

**Authors:** Eva Ortega-Paino, Eduardo Oliver

**Affiliations:** ^1^Centro Nacional de Investigaciones Oncológicas (CNIO), Madrid, Spain; ^2^Centro de Investigaciones Biológicas Margarita Salas (CIB-CSIC), Madrid, Spain

**Keywords:** science diplomacy, scientific diaspora, brain-connection, science advisory bodies, brain drain/gain

## Abstract

RAICEX (Red de Asociaciones de Investigadores y Científicos Españoles en el Exterior), the Network of Associations of Spanish Researchers and Scientist Abroad, consists of more than 4,000 Spanish researchers distributed in 18 countries in 5 different continents. RAICEX was established in July 2018 by 15 foundational members: the associations of Spanish Researchers in the USA, México, Ireland, Sweden, Denmark, France, Italy, Japan, Australia, China, UK, Germany, Switzerland, Belgium, and Norway. Since then, 3 more associations have joined: Emirates, Netherlands and South Africa. RAICEX was born with the main goal: “*promoting the exchange of experiences and knowledge between Spanish researchers and scientists abroad and all the stakeholders of the Spanish System of Science, Technology and Innovation (SECTI), serving as an advisory body, information channel and catalyst for international relations in scientific matters, contributing to the progress of science*.” Their main objectives are: (1) to provide support to researchers and scientists in mobility and personal development, offering training, information and guidance, as well as providing contact with all the other associations that make up the global network; (2) to disseminate and give visibility to the value of Science and the work of researchers and scientists, promoting communication of the advances of knowledge in all areas of society; (3) to promote international relations and cooperation between researchers / scientists and public and private organizations, from a global perspective; (4) to share the acquired knowledge and experience in different research and science systems abroad to advise, provide feedback and contribute to the progress of the whole SECTI. In this Case Study a particular scenario of the Spanish scientific diaspora, including history, reasons for going abroad, and consequences for the Spanish R&D system, shall be introduced to readers. The impact that RAICEX and its foundational members have had in the Spanish National System since the creation of the first community in the UK by 2012 will also be discussed. RAICEX's activities range from providing advice to newcomers and carrying out science dissemination, to becoming an advisory body to governments and institutions. The Spanish scientific diaspora is an extensive network committed to cooperation and brain connection.

## Introduction: The Global Scientific Diaspora

It is well-documented that in all countries there is a balance between the scientists that leave the country and the international researchers that the country takes in. This process has occurred as the result of transnational academic mobility since the albors of science. However, when this equilibrium is negatively balanced there could be a relevant loss of scientific talent for the country (Salgado, 2016; Cavallini et al., 2018). In this regard, in 1963 the Royal Society coined the term brain-drain to define the emigration of scientists, technologists, academics and many other high-level professional groups, to obtain better salaries, equipment or conditions (Royal Society, 1963). This fact resulted in an unprecedented social and political debate, which made the United Kingdom change its policies for investment, attraction, and retention of talent (Balmer et al., 2009).

The scientific diaspora has always existed and will always exist, although it reached its peak in many countries at the beginning of the twenty-first century, as developed countries wanted to incorporate foreign knowledge and talent generating wealth and an improved economic competitiveness. However, it was more recently, in 2011, when the Royal Society published the report “Knowledge, Networks and Nations: Global Scientific Collaboration in the twenty-first Century” (Royal Society, 2011) and defined the characteristics of the current global scientific diaspora. This study provided an overview of the global scientific landscape and showed the growing importance of research collaboration. The report concluded with five main recommendations that can be summed up in one: international science and collaborations must continue to be supported and promoted to address global challenges—a shift toward brain linkage or connection. Something that, 10 years later, still seems to be more fashionable than ever. Today science is understood—or should be understood—as a global phenomenon in which mobility of talent and international collaboration play a key role. In this regard, the report also highlights the fact that there are more than seven million researchers around the world who want to collaborate with the best professionals in their field to seek new knowledge and to make progress (Royal Society, 2011). Indeed, the so-called global scientific diaspora was the subject of study in the journal *Nature* in 2012 (Van Noorden, 2012). This study shows that, when researchers move abroad, they mainly have professional development in mind and to achieve this they seek the most suitable environments in terms of training, critical mass, and resources. Scientists, in their search for intellectual and scientific prosperity, move to countries with a consolidated R&D system and with solid foundations that offer them long-term guarantees. These systems are dynamic and flexible, with well-defined professional projection paths and great transverse mobility—across institutions, regions, fields of knowledge and sectors. Added to this is the fact that scientists are attracted to systems in which the research career is based on meritocracy, which guarantees scientific excellence and a powerful professional group made up of the best researchers in the host country. On the other hand, scientists also emigrate due to lack of opportunities in their countries of origin (Suresh, 2012; Cavallini et al., 2018). This exit is positive when individuals educated abroad are capable of reverting what they learned abroad to the country in which they began their educational development, or when the foreign “brains” come to nourish the gap left by those who moved out. This has been called brain circulation, and it is something that other countries know very well (Johnson and Regets, 1998; Kone and Ozden, 2017; Yu, 2021). In addition, encouraging brain connection between countries has proved to be useful for Science and multilateral collaborations between countries (Meyer and Brown, 1999; Gamlen, 2006; Balmer et al., 2009; Kone and Ozden, 2017; Yu, 2021).

## Context: Spain's Particular Scenario

The relevance of scientific mobility phenomena and brain-drain was early understood by governments in certain countries that established transnational mobility policies allowing them to attract talent and diversity. This was translated into economic prosperity thanks to a proper balance between the talent emigrated, brain-drain, and the talent attracted or retained, brain-gain, as it is the case of Sweden with its well-known and established programs from the Swedish Research Council (Vetenskaprådet, 2018). This ratio is an important diagnostic marker of any country's economic health, since the most developed countries invest a significant percentage of their GDP in science, research, development and innovation to maintain this balance. This effect can be seen with other examples, such as that of the Scandinavian countries, where both Sweden and Denmark invest over 3% of their GDP, a figure that falls short when compared to those of countries such as Israel and South Korea, which are close to 5% (OECD Data, 2000–2020). In other countries such as Spain, a modest 1.25% at the end of 2020, similar to what was achieved by the end of a domestic economic boom in mid 2006, according to the National Institute of Statistics (INE, 2020), translates into a slowdown of what should be the core of the country' machinery: research, development and innovation (R&D&i). Indeed, Spain has traditionally been a country with a very low investment of its GDP in R&D&i, having reached its maximum level of 1.36% in 2010, still far by more than one point from the world average, which is close to 2.5% (OECD Data, 2000–2020; INE, 2020).

After the 2008 Great Recession, Spain plunged into a major economic crisis. This worldwide economic crisis, which originated a year earlier in the United States, was mainly due to subprime mortgages that led to the bankruptcy of Lehman Brothers. This crisis strongly affected the economy of the European Union, whose states had accumulated huge amounts of debt, orchestrated under the pressure of Germany which was also tremendously hit by the crisis, but from which it has been recovered without digression (Szczepanski, 2019).

Nevertheless, in Spain, the crisis had an additional internal aspect, mainly due to an unprecedented real estate bubble, at the level of both the construction business and the financial system. The high unemployment level in the country led to a massive outflow, not only of immigrants who had come to work in construction during the previous years, but also of young people who were forced to leave (Royo, 2020), despite their being, most likely, the best educated generation in our history. This brain-drain forced many young people to emigrate to traditional “embracing” countries such as the US and the UK, but also to new destinations such as Sweden, Switzerland, Denmark, France, Germany and even Mexico, where opportunities for development and a stable future were greater (Van Noorden, 2012; Salgado, 2016). This emigration of labor reached all sectors and reduced assets in the Spanish Science and Technology system.

As already pointed out, one of the reasons for scientists to emigrate is the lack of opportunities in their countries of origin—something that happened with special emphasis in Spain. If we take a look at the situation in other countries, such as the United Kingdom, there is almost a balance between researchers who leave the country for destinations such as the US, Canada and Australia, mainly, and those received from these countries, mainly Italy and Germany. This is also the case in Germany, where the balance is even more obvious. In 2012 the percentage of researchers who left Germany was 23%, similar to the percentage of international researchers who chose this country as their destination according to data from the GlobSci survey (Van Noorden, 2012). Spain, in that year, showed a similar pattern to Germany's, but with a percentage three times lower and with an international immigration profile of countries where the language was close to Spanish, such as France and Italy, or exactly the same, in the case of Argentina (Van Noorden, 2012). The study published in *Nature* also highlights that the three countries chosen by Spanish scientists as the main destinations were, and still are, the US, the UK and Germany, followed by Japan, France, Australia, and the rest of the EU (Van Noorden, 2012; Salgado, 2016).

Today we still lack accurate, updated figures about emigration in the R&D sector. According to the OECD, in 2011 there were around 12,000 Spanish researchers abroad (OECD, 2013). But that number increased in subsequent years. In fact, according to 2015 data from the British Higher Education Statistics Agency, in British universities alone, there was a 40% increase in the total number of Spanish researchers compared to 2012 (HESA, 2022). Therefore, on the basis of the same assumption, we can say that the global number of Spanish researchers abroad reached around 20,000 in the following years, almost 0.1% of the total emigration, specialized in science, technology and research in general—which represents around 10% of the workers in the sector (OECD, 2013). These figures are still striking, and this is why the word “exodus” (or rather we should call it exile) is often mentioned in different forums, not only regarding thousands of young, and not so young, people who have left our country, in many cases taking their families with them and facing a rather uncertain future with slim chances of return (Ortega-Paíno and Oliver, 2021).

## The Rise of Spain's Organized Scientific Diaspora

The loss of critical mass in Spain, due to the aforementioned crisis and the fact that Spaniards started to improve their language skills in English mainly, is followed by another immediate consequence: an increased diaspora. A good example of this, after the 2008 crisis, is the birth of associations of scientists and researchers working in other countries. This associative movement, born in the United Kingdom between 2011 and 2012, inspired other countries and as a result, in the following years, the Spanish scientific diaspora became organized (Catanzaro, 2012).

Since that date, 18 associations of Spanish scientists (plus 1 recently set up in Chile and 2 more in the process of constitution, in Canada and Brazil) have been born in five continents, which up to date represent more than 4,000 “brains” (Table 1). These grass-roots citizen associations are independent, non-profit, non-partisan, but not apolitical scientific societies—since one of their main purposes is to work on policies that may improve the situation of their origin country's R&D&I system. These organizations have been conceived and orchestrated by scientists eager to maintain ties with Spain, promoting multilateral relations and bringing the experiences of other countries closer together (Melchor, 2016; Oliver, 2016).

**Table 1:** The 18 associations of Spanishs scientist and researchers abroad officially launched from 2012 to 2020.

**Association name and acronym**	**Host country**	**Year of registry**	**Website**
Sociedad de Científicos Españoles en Reino Unido (SRUK/CERU)	United Kingdom	2012	www.sruk.org.uk
Españoles Científicos en USA (ECUSA)	United States	2013	www.ecusa.es
Sociedad de Científicos Españoles en la República Federal de Alemania (CERFA)	Germany	2014	www.cerfa.de
Asociación de Científicos Españoles en Suecia (ACES)	Sweden	2014	www.aces-sffs.com
Científicos Españoles en Dinamarca (CED)	Denmark	2014	www.ced-sfd.org
Spanish Researchers in Australia-Pacifico (SRAP)	Australia	2015	www.srap-ieap.org
Asociación de Científicos Españoles en Japón (ACEJapon)	Japan	2016	www.acejapon.jp
Asociación de Investigadores Españoles en la República Italiana (ASIERI)	Italy	2016	www.asieriitalia.altervista.org
Spanish Research Society in Ireland (SRSI)	Ireland	2017	www.srsireland.org
Red de Científicos Españoles en México (RECEMX)	Mexico	2018	www.recemx.com.mx
Científicos Españoles en Bélgica (CEBE)	Belgium	2017	www.cebebelgica.es
Red de Investigadores China-España (RICE)	China	2017	www.ric-e.net
Asociación de Científicos Españoles en la Confederación Helvética (ACECH)	Switzerland	2018	www.acech.ch/
Sociedad de Investigadores Españoles en Francia (SIEF)	France	2018	www.siefrancia.com
Asociación de investigadores españoles en Noruega (IENO)	Norway	2018	www.sfnoieno.wordpress.com
Científicos Españoles en Países Bajos (CENL)	Holland	2019	www.cenetherlands.nl
Asociación de Científicos Españoles en Sudáfrica (ACE Sudáfrica)	Sudafrica	2019	acesudafrica.wordpress.com
Asociación de científicos e investigadores españoles en Emiratos Árabes Unidos (ACIEAU)	United Arab Emirates	2020	www.acieau.es

These grass-roots associations were set up with several objectives: to become an efficient network that connects Spanish researchers in the country of destination and supports newcomers; to disseminate science at all levels of society, actually raising awareness of the relevance of their progress and the importance of investing in them; to take science where it is least expected in its desire to disseminate, but also organize seminars and symposiums with the most prestigious Spanish researchers and introduce them in their destination countries; to dedicate efforts to build bridges of international collaboration between groups, associations and institutions bilaterally and multilaterally; to sign agreements with leading universities and research centers to encourage joint participation in European projects; to facilitate the exchange of researchers between countries and carry out the task of mentoring to advise those who are emigrating in their formative thinking (Melchor, 2016; Oliver, 2016).

In a clear work of Science diplomacy, this probably being the best of the consequences that the brain-drain has been able to revert to Spain, our assets abroad act as advisory bodies to Spanish public and private organizations, sharing the knowledge and experience of thousands of professionals from different fields of science or presenting reports, recommendations, and critical analyses with proposals for improvement (RAICEX News, 2021a). Many of these scientists have lived in multiple countries, have years of experience abroad, and are extremely knowledgeable about the work in R&D systems in some of the world's leading countries. For all these reasons, researchers and governments understood that maintaining good contact with the scientific diaspora—a brain-connection approach- could be highly beneficial since they represent an asset for Science Diplomacy and a way of recovering lost talent (Gamlen, 2006; Elorza Moreno et al., 2017; Kone and Ozden, 2017).

## Details of the Case Study: Raicex, the Network of Associations of Spanish Scientists and Researchers Abroad

**Figure 1:**
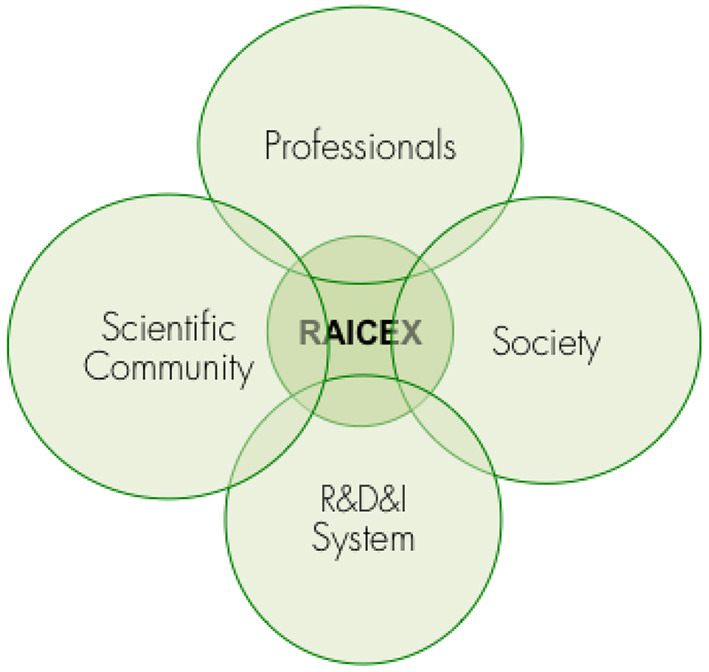
The impact of RAICEX's activities and objectives.

**Figure 2:**
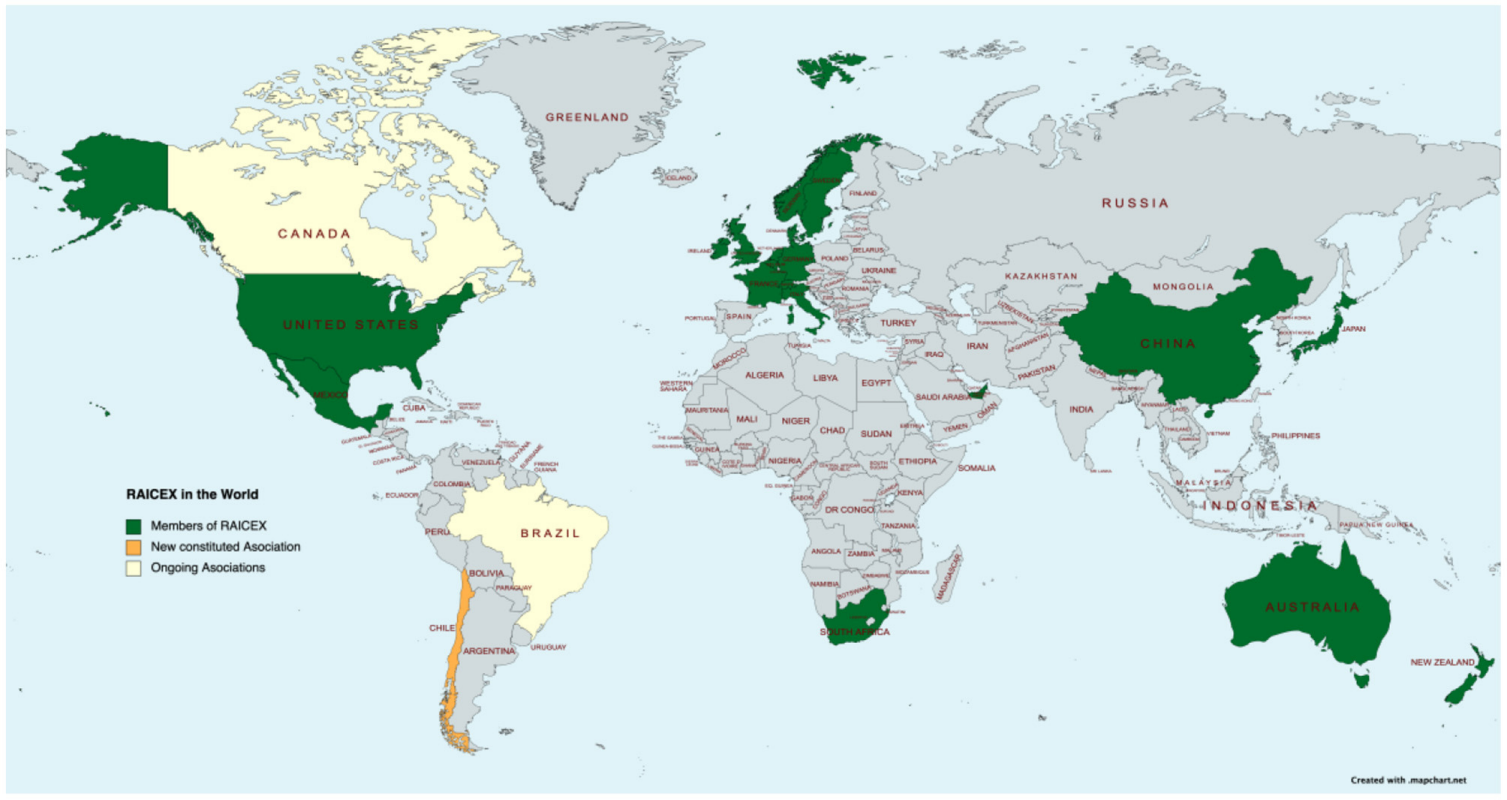
The world-wide presence of RAICEX members. Green color represents active members; Orange color represents new constituted associations yet to sign the incorporation agreement; Yellow color represents incoming new association. The figure represents data from 2012 to 2022.

In July 2018, the representatives of 15 of these associations of Spanish scientists and researchers abroad—the ones existing at that time—joined efforts in a unique voice and founded the so called network of associations RAICEX (Red de Asociaciones de Investigadores y Científicos Españoles en el Exterior) (SINC, 2018). RAICEX is an independent, non-profit organization made up of 18 associations registered in different countries (Table 1), which was born in response to the growing need to unite and represent the community of Spanish scientists and researchers abroad, under a common framework. Their general interest is to transmit and share the skills and knowledge acquired in a global scientific context and multilateral collaboration (Figures 1, 2). RAICEX's mission is to promote the exchange of experiences and knowledge between Spanish researchers and scientists abroad and all the stakeholders of the SECTI, serving as an advisory body, channeling information and catalyzing international relations multi-directionality in scientific matters, thus contributing to the progress of science. The global objective of RAICEX is to generate a single voice that, respecting the independence of each association, encompasses Spanish scientists and researchers abroad in a common forum, pursuing the following objectives: (1) In the training of Researchers and Scientist: to support researchers and scientists in terms of mobility and professional development, providing training, information and guidance, as well as facilitating contact with all associations; (2) In communication with Society: to disseminate, give prestige and visibility to the value of Science and the work of researchers and scientists, while promoting the communication of scientific and technological advances in all areas of society; (3) In the internationalization of the Scientific Community: to favor international relations and cooperation between researchers/scientists, public and private organizations and bodies, from a global perspective in matters of research, science and technology, thus promoting networking; (4) In advising SECTI: to share the experience and knowledge acquired in the different research and science systems abroad to advise, provide feedback and contribute to the progress of SECTI as a whole (Figure 1) (RAICEX, 2018).

RAICEX is made up of its main body, the Assembly of Members (AoM), in which the 18 countries (Figure 2) are represented by their legal body, usually their presidents. The AoM elects their Managing Committee. This Committee is the decision-making body of the network and is also in charge of electing the Secretary General (SG) and the External Advisory Board (EAB). The Managing Committee also decides the *ad-hoc* commissions needed for the proper functioning of the network. Currently, RAICEX has 3 commissions working on science policies and diplomacy, gender equality and communication. The Secretary General, represented by a managing member from any of the associations who has returned back to Spain, performs the role of Public Affairs Officer of the network and represents the network in Spain wherever required (RAICEX, 2018). This role has allowed direct interaction with the main bodies or stakeholders of the Spanish scientific landscape such as the Ministry of Science, Technology and Innovation, the Spanish Agency for International Development Cooperation (AECID), the Spanish Foundation for Science and Technology (FECYT) and many other actors both in the public and the private sector.

## Discussion

Since RAICEX was set up, the network, as well as its activities, have increased in number and impact. The participation of RAICEX as an advisory body for science policy and diplomacy has been growing and, RAICEX has been involved in the Amendment to the Science Act 14/2011 (RAICEX News, 2022a), a public call where members of the SECTI can submit comments and recommendations to improve the law. In line with this, RAICEX was previously invited to present their views on the new Science Act and the National Pact for Science and Technology in front of the Science committee of the Spanish Congress of Deputies (RAICEX News, 2021b). At present, RAICEX is (1) collaborating with the main public and private stakeholders, such as the Ministry of Science, Technology and Innovation, the Ministry of Foreign Affairs, the Ministry of Migrations as well as Instituto Cervantes, the Royal Academy of Sciences, the Spanish Association for Biotechnology (ASEBIO), Farmaindustria, Fenin, among others, to draw up and elaborate policies for attraction and retention of domestic as well as international talent (data extracted from RAICEX website). This will be reflected in the ATRAE (Attraction and retention of talent to Spain) report, an initiative of RAICEX. This report is based on ten main commandments and developed by the scientific policy and diplomacy commission (RAICEX Science Policy Talent Attraction committee, 2019). The extended version of this report is expected to be publicly presented in the autumn of 2022; (2) giving visibility to their diaspora by collaborating with the Spanish Broadcasting Corporation (RNE) and performing weekly interviews with their members abroad, as well as in other radio programmes and newspapers; (3) participating in mentoring programmes such as Researchers Beyond Academia (REBECA), a mentoring programme for researchers who want to explore careers beyond the academic pathway organized by EURAXESS Spain (FECYT, 2021); (4) working on gender inequality matters and collaborating with other associations and universities to study and narrow the existing gender gap in science and technology. This commission has developed videos to show this gap and raise awareness within society (RAICEX Research Gender committee, 2022); (5) organizing scientific meetings, such as the series Bridging European Science within the Nordic Countries Associations, among others; and last but not least, (6) building bridges in cancer research through collaborations with a founding agent, CRIS cancer, by providing grants to Spanish researchers who later pend a couple of months back in Spain, therefore, favoring talent attraction (RAICEX News, 2022b). These examples represent only the most relevant and recent achievements that can be extracted from the RAICEX website.

In recent years, many voices from groups of researchers and scientists have demanded changes within the SECTI. Many of these voices, and those gathered abroad in associations of researchers and scientists in particular, came together in 2018, as mentioned above, in a single voice channeled by RAICEX. From this group, as well as from the same national researchers and other organizations, concerns have been expressed, proposals have been presented, mobilisations and demonstrations have been organized under the slogan *#SinCienciaNoHayFuturo (No Science, No Future)*, to position Spain in the international frontline of Science and Innovation. A task that, without being pessimistic, seems quite complicated if we take into account that, traditionally, science has never been the main character in the play (Segurola, 2014).

Echoing these voices, in July 2020 and after more than a decade of continuous cuts, the Government, in an attempt to revive our R&D&i system as if it was an intensive care unit, presented in Moncloa the Shock Plan for Science, with short-term measures that would serve as a hinge for the future “Investment and Reform Plan for the Recovery of the Economy.” The Plan encompasses three basic axes: investment in research and health; the transformation of the science system and the attraction and retention of talent and the promotion of business and industrial R&D&i in science (Moncloa, 2020). This Shock Plan was followed by proposals such as the National Pact for Science and Innovation, insistently demanded by many scientific groups, and which has been joined by 86 entities, included RAICEX (RAICEX News, 2020). The objectives of this long-awaited Pact are focused on Resources, the System and People. There is no doubt that these three ingredients are essential in the master recipe for European investment under the Recovery, Transformation and Resilience Plan, which the Government had expected as if it was manna.

As of today, the actors involved in Science and Innovation are looking forward to the development of the draft bill proposal that has already been presented to the Congress of Deputies and on which an *ad-hoc* subcommittee is already working. This preliminary project is still supported by three basic pillars: the scientific career, transfer to society and governance. Three pillars that, as a master formula, include the need to increase the number of R&D&i assets, to attract emigrated talent, to increase public-private collaboration and, of course, to maintain everything orchestrated under a governance that facilitates the coordination of this cast in science (Ministerio de Ciencia e Innovacion, 2022).

When looking overseas, RAICEX (and its members) is not the only organized scientific diaspora in the world. In fact, students, investigators and graduated Portuguese workers of all areas in science are represented by the Portuguese Association of Researchers and Students in the United Kingdom (PARSUK) a network that promotes the integration, collaboration, and development of their members (PARSUK, 2008). Similar to this, Polish research diaspora is organized under the umbrella of Polonium Foundation, an independent non-profit aiming at turning Polish brain drain into brain circulation (Polonium Foundation, 2016). As a matter of fact, since 2016 EURAXESS organize an annual meeting of European scientific diasporas in North America form which it publish a report with views and analysis from the speakers that can be useful for governments (EURAXESS, 2016). Also recently analyzed in this issue, the diaspora organizations from Latin America and the Caribbean has been found to be a tool to engage with governmental and non-state actors and are active science diplomacy stakeholders promoting the scientific developments of their country or their researchers, as well as enabling access to research resources creating alliances for scientific, institutional, and academic collaborations (Figueroa et al., 2022). Nevertheless, there are some aspects that make RAICEX unique if compare to other diasporas: (a) it is a bottom up initiative created by the own scientists to collaborate and connect with Spanish public and private scientific institutions; (b) it is a big network of networks, since it represents a big number of associations which are independent themself allowing them to perform their own activities; (c) it is present in more than eighteen countries within five continents while having a general secretary head quarter in Spain which boost connections with institutions and organizations in the country; (d) it serves as an umbrella to boost the power of representation of its association members beyond Spanish institutions while the associations play an important role connecting the network world-wide.

As RAICEX grows—the association in Chile has been recently set up and others, in Brazil and Canada, are on their way—there are new goals to achieve in providing the network with a proper, effective structure. These goals could be summarized in: (1) building the network of RAICEX ambassadors to give support geographically to the Secretary General by increasing the participation of the network in as many activities and meetings with its stakeholders as possible in all Autonomous Regions in Spain; (2) creating an *ad-hoc* commission for fundraising; (3) acting as an advisory board and channel to as many stakeholders as possible in developing policies that could help those scientists and researchers wishing to return to Spain; and (4) interacting and advising our embassies abroad in science and technology-related issues and topics for solving global challenges that could arise, as has been the case with the Covid-19 pandemic.

## Conclusions

The need for organizing the diaspora has now been established. The Spanish Scientific Diaspora, set up in 18 different countries, with 4 more under way on-going, and acting under the umbrella of RAICEX, is a clear example of success that can help to become an advisory body to governments and institutions, by advising newcomers and carrying out science dissemination. The Spanish Scientific Diaspora represented by RAICEX is an extensive network committed to cooperation and brain connection. Being connected and listening to demands from scientists, not only inside but outside the country, can contribute to providing us with a more attractive system capable of attracting and retaining domestic and international talent.

## Author Contributions

EO-P and EO equally contributed to interpreting this case study. Both authors contributed to the article and approved the submitted version.

## Conflict of Interest

EO-P is the current General Secretary of RAICEX and ambassador of ACES in Spain. EO is the former General Secretary of RAICEX and ambassador of SRUK in Spain.

## Publisher's Note

All claims expressed in this article are solely those of the authors and do not necessarily represent those of their affiliated organizations, or those of the publisher, the editors and the reviewers. Any product that may be evaluated in this article, or claim that may be made by its manufacturer, is not guaranteed or endorsed by the publisher.
